# Neural Coding of Perceived Odor Intensity^1,2,3^

**DOI:** 10.1523/ENEURO.0083-15.2015

**Published:** 2015-12-03

**Authors:** Yevgeniy B. Sirotin, Roman Shusterman, Dmitry Rinberg

**Affiliations:** 1 The Rockefeller University, New York, New York 10065; 2 Janelia Farm Research Campus, Howard Hughes Medical Institute, Ashburn, Virginia 20147; 3 Sagol Department of Neurobiology, University of Haifa, Haifa 34905, Israel; 4 NYU Neuroscience Institute, NYU Langone Medical Center, New York, New York 10016; 5 Institute of Neuroscience, University of Oregon, Eugene, OR 97405

**Keywords:** Concentration versus adaptation, extracellular electrophysiology, human psychophysics, olfactory bulb

## Abstract

Stimulus intensity is a fundamental perceptual feature in all sensory systems. In olfaction, perceived odor intensity depends on at least two variables: odor concentration; and duration of the odor exposure or adaptation. To examine how neural activity at early stages of the olfactory system represents features relevant to intensity perception, we studied the responses of mitral/tufted cells (MTCs) while manipulating odor concentration and exposure duration. Temporal profiles of MTC responses to odors changed both as a function of concentration and with adaptation. However, despite the complexity of these responses, adaptation and concentration dependencies behaved similarly. These similarities were visualized by principal component analysis of average population responses and were quantified by discriminant analysis in a trial-by-trial manner. The qualitative functional dependencies of neuronal responses paralleled psychophysics results in humans. We suggest that temporal patterns of MTC responses in the olfactory bulb contribute to an internal perceptual variable: odor intensity.

## Significance Statement

Establishing a link between perception and neural activity is one of the major goals of systems neuroscience. Yet, tracking perceptual variables in animal models, where one can perform neural recording, remains a challenge. Here we demonstrate a consistency between human perception of odor intensity and activity of mitral/tufted cells (MTCs) recorded in the olfactory bulb of awake mice as a function of two physical variables: odor concentration and the duration of odor exposure. Human perception of odor intensity decreased sharply after just one sniff of odor. Consistently, sniff-locked MTC odor responses changed abruptly after the first sniff so as to mimic responses to lower odor concentrations. We suggest that early processing stages may already contribute to an odor intensity percept.

## Introduction

One of the major aims of systems neuroscience is to link neural activity at different stages of information processing with specific aspects of perception. Strong links with perception have been established in the visual and somatosensory systems (Britten et al., 1992; Johnson et al., 2002; Romo et al., 2002); however, such perceptual links are dramatically absent in olfaction (but see Wilson and Stevenson, 2006). Despite this, the olfactory system has become an established model for studying neural coding due to its relatively simple, accessible, and evolutionarily conserved organization (Hopfield, 1995; Laurent, 2002; Kepecs et al., 2006; Wilson and Mainen, 2006).

Perhaps the most basic perceptual axis for all senses is stimulus intensity. Intensity is a perceptual variable that facilitates comparisons of different objects within a single modality as well as across modalities (Over and Mackintosh, 1969; Marks, 1978; Wojcik and Sirotin, 2014). In olfaction, intensity is a common feature of all odors (Beck et al., 1954; Engen, 1964), and the perceptual organization of intensity is conserved across the mammalian species (rats and humans). Intensity is related to odor concentration as a power function (Cain, 1969; 1970; Moskowitz et al., 1976; Wojcik and Sirotin, 2014), and intensity discrimination performance is scale invariant (Stone, 1963; Stone and Bosley, 1965; Wojcik and Sirotin, 2014). Even the relationship between intensity and the physicochemical properties of odors appears to be conserved across species (Edwards and Jurs, 1989; Wojcik and Sirotin, 2014). The conservation of the perceptual properties of intensity in olfaction likely reflects the highly conserved neural-processing mechanisms of olfactory systems across species. While it has been shown that neuronal activity in the piriform cortex, entorhinal cortex (Rolls et al., 2003), and amygdala (Anderson et al., 2003) correlate with intensity perception, how neural activity at specific stages in olfactory processing contributes to this perceptual variable is unclear. In rats and humans, odor intensity grows systematically with concentration and rapidly decreases with adaptation (Engen, 1964; Ekman et al., 1967; Cain, 1970; Pryor et al., 1970; Steinmetz et al., 1970; Wojcik and Sirotin, 2014; Cain and Engen, 1969). Thus, perceived intensity for a given odor is a function of at least two variables, as follows: the physical odor concentration and the sampling duration. Therefore, in order for a neuronal response to underlie odor intensity coding, it should change consistently with concentration and sampling duration. In the current work, we will exploit this consistency in order to reveal the relationship between neuronal responses and a perceptual variable.

Mitral/tufted cells (MTCs) in the olfactory bulb have been a subject of multiple studies due to their central role in the processing of olfactory information. MTCs are the only cells that transmit information from the bulb to higher brain areas. They receive primary input from individual glomeruli, and their processing is affected by other glomeruli via lateral interactions. In awake animals, where the dynamics of these cells are very different from those in the anesthetized state (Rinberg, 2006; Kato et al., 2012), olfactory information is encoded by MTC activity at sub-sniff timescales (Cury and Uchida, 2010; Shusterman et al., 2011). Moreover, recent work demonstrated that these fine temporal patterns can be read by higher brain areas (Smear et al., 2011, 2013), thus establishing a connection between coding properties of MTCs and their role in behavior. Our knowledge about concentration and adaption dependencies of these cells is mostly based on recordings from anesthetized animals (Chalansonnet and Chaput, 1998; Wilson, 1998; but see Patterson et al., 2013). Here we explore both concentration and adaptation dependencies of MTCs in awake mice and their potential role in forming the intensity percept.

In mammals, the flow of odor to the olfactory epithelium is controlled by the breathing/sniffing rhythm (Kepecs et al., 2006). This rhythm sets the natural time scale of odor processing to the duration of a single inhalation/exhalation (“sniff”) cycle. Based on experiments in rodents, the structure and the temporal scale of information encoding in the olfactory system (Cury and Uchida, 2010; Shusterman et al., 2011) is consistent with behavioral results that one to two sniffs are sufficient for olfactory decision making (Uchida and Mainen, 2003; Abraham et al., 2004; Rinberg et al., 2006). Here we compare concentration and adaptation dependencies of MTCs with human odor intensity perception, both measured on sniff-based time scales.

## Materials and Methods

### Neural recording

#### Animals

Data were collected in four C57BL/6J mice. Mice were 6–8 weeks old at the beginning of behavioral training and were maintained on a 12 h light/dark cycle (lights on at 8:00 P.M.) in isolated cages in a temperature- and humidity-controlled animal facility. All animal care and experimental procedures were conducted in strict accordance with a protocol approved by the authors’ Institutional Animal Care and Use Committee.

#### Electrophysiology

MTC spiking activity was recorded using 32-channel Si-probes [model a4x8-5mm-150-200-312 (H32), NeuroNexus]. Cells were recorded in both ventral and dorsal mitral cell layers. The identity of MTCs was established on the basis of criteria formulated in previous work (Rinberg, 2006). The data were acquired using a 32-channel data acquisition system (Digital Lynx, Neuralynx) with widely open broadband filters (0.1–9000 Hz) and sampling frequency of 32.556 kHz.

#### Sniff recording

To monitor the sniff signal, we implanted a thin 7-mm-long stainless cannula (23 gauge capillary tubing, Small Parts) in the nasal cavity. The cannula was capped between experimental recordings. During experiments, the cannula was connected to a pressure sensor with polyethylene tubing (801000, A-M Systems). The pressure was measured with a pressure sensor (MPX5050, Freescale Semiconductor) and a homemade preamplifier circuit. The signal from the preamplifier was recorded together with electrophysiological data on one of the data acquisition channels. The sniff monitor was calibrated against a known flow as described by Shusterman et al. (2011). The lag between the pressure zero-crossing and airflow velocity zero-crossing was <1 ms.

#### Surgery

Mice were anesthetized using isoflurane gas anesthesia. The horizontal bar for head fixation, pressure cannula, and electrode chamber were implanted during a single surgery. To implant the sniffing cannula, a small hole was drilled in the nasal bone, into which the cannula was inserted and affixed with glue, and stabilized with dental cement. To implant the electrode chamber, a small craniotomy (∼1 mm^2^) was performed above the left or right olfactory bulb. After the insertion of the Si-probe, the electrode chamber was fixed by dental cement to the skull, posterior to the olfactory bulb. The reference electrode was implanted in the cerebellum. The mice were given at least 5 d after a surgery for recovery.

#### Behavioral procedure and training

After recovery, the mice were placed in the head-fixation setup. The first few sessions were brief (10–20 min) and served to acclimate the animals to head fixation in the setup. Mice typically remained mostly quiescent after one to two sessions of head fixation, after which odor sessions started. We delivered one of four odors at three concentrations in a pseudo-random sequence with an average interstimulus interval of 7 s and a stimulus duration of at least 2 s. One session usually lasted for ∼1.5 to 3 h and contained 600-1200 trials (50–100 trials per stimulus).

#### Odor delivery

For stimulus delivery, we used a nine-odor air dilution olfactometer. The airflow through the selected odorant vial was diluted 10 times by the main airflow stream and homogenized in a long thin capillary before reaching the final valve. It took approximately 500–1000 ms to prepare the homogenized mixture and equilibrate the concentration. A steady stream of 1000 ml/min of clean air was flowing to the odor port at all times except during stimulus delivery, when the flow from the olfactometer was directed to the odor port. After sufficient mixing and equilibration time, the final valve (four-way Teflon valve, NResearch) switched the odor flow to the odor port, and diverted the clean airflow to the exhaust. All flows and line impedances were tuned to minimize the pressure shock resulting from line switching and the time of odor concentration stabilization after opening the final valve. The temporal odor concentration profile was checked by mini-PID (Aurora Scientific). The concentration reached a steady state ∼40 ms after final valve opening.

Odor delivery was triggered on the end of the inhalation phase of the sniff cycle, which was detected by positive-going zero-crossings of the pressure signal. This prevents odor from being delivered at random times during inhalation, which would confound our analysis. Furthermore, because no odor enters the nose during the exhalation phase, this allows enough time for the odor stimulus to reach a steady state of concentration by the time the animal begins inhaling.

We used multiple odorants obtained from Sigma-Aldrich. The odorants were stored in liquid phase (diluted 1:5 in mineral oil) in dark vials. The odorant concentration delivered to the animal was reduced an additional 10-fold by air dilution. The following odorants were used: acetophenone, amyl acetate, benzaldehyde, butyric acid, decanol, ethyl acetate, ethyl tiglate, 1-hexanol, hexanoic acid, hexanal, 2-hexanone, hexyl acetate, R-limonene, isopropyl tiglate, methyl benzoate, methyl salicylate, 1-octanol, and 2-undecanone.

All of the analyses discussed below were performed in MATLAB (MathWorks).

#### Spike extraction

Acquired electrophysiological data were filtered and spike sorted using a WaterShed software package written by Alexei Koulakov (Cold Spring Harbor Laboratory, Cold Spring Harbor, NY).

#### Temporal warping

Sniffing recordings were down-sampled to 1 kHz, and filtered in the range of 0.5–20 Hz. Initially, the times of inhalation onset and offset were detected by negative and positive zero-crossings, respectively. Often the positive zero-crossing at the end of inhalation phase was not well defined, owing to the very shallow slope of the signal. To more reliably estimate the offset of the inhalation phase, we fit a parabola to the minima of the pressure signal following the onset of the inhalation (Shusterman et al., 2011). Inhalation offset was defined as the second zero-crossing of the parabola. We defined the following two intervals: the first is from inhalation onset to inhalation offset, and the second is the rest of the sniffing cycle, from the inhalation offset to the next inhalation onset. For the whole session, we estimated an average duration for both intervals. Each interval of the sniffing data, together with correspondent spiking data, was stretched or compressed to make its duration equal to the duration of the average interval. For analysis, we used only sniffs of typical duration (between 200 and 500 ms), which constitute ∼80% of all sniffs. Analysis of odor responses was restricted to the first 200 ms of response following sniff onset in warped time coordinates.

#### Odor responses

**Figure 1.**
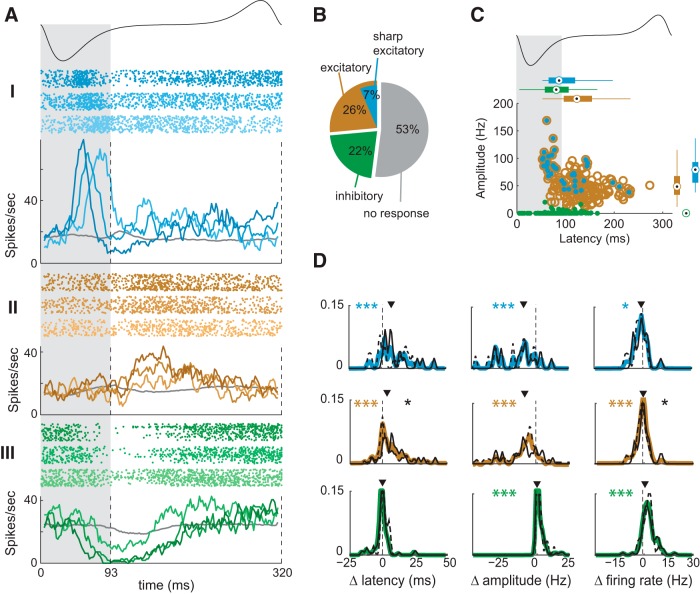
MTC responses change with odor concentration. ***A***, Sniff-warped raster and PSTH plots of sharp excitatory (I-cyan), excitatory (II-brown), and inhibitory (III-green) responses of individual MTCs for 3-fold and 10-fold changes in odor concentration (shown as color shades). Top, Schematic sniff waveform. Gray shading, Inhalation; gray trace, activity of the MTC during blank sniffs. Vertical dashed lines indicate the beginning and end of inhalation interval. ***B***, Distribution of different response types observed in the data. ***C***, Scatter plot comparing amplitude and latency of sharp, excitatory and inhibitory responses (color notations as in ***B***). Boxplots show marginal response distributions: circle is median, thick line is the IQR, thin lines on either side extend to 1.5 × IQR beyond the 25% and 75% quartiles or the farthest data point, whichever is smaller. ***D***, Normalized distributions of changes of latencies (left column), amplitude (central column), and firing rate (right column) with a threefold concentration change across cells for different response types (color notations as in ***B***). Colored asterisks denote significance of test for zero median (*p < 0.05, **p < 0.01, ***p < 0.001; Wilcoxon rank sum test). Black solid and dashed lines show distributions of response latencies for early (<100 ms) and late (>100 ms) responses respectively. Black asterisks denote significant differences between two distributions. Arrows mark the position of the median.

We compared the distributions of the neuronal activity with and without odors. Neuronal activity without odor was sampled from sniffs preceding odor delivery across all trials. Neuronal activity for a given odor was sampled from the first sniff after stimulus onset for the trials containing a correspondent odor delivery. Units were considered responsive if their spike probability statistically differed from the distribution of baseline responses (randomly subsampled) in at least one 10 ms bin relative to inhalation onset (*p* < 0.005) or if their average spike rate over the sniff cycle differed significantly from baseline (*p* < 0.05). Responses were considered initially excitatory (inhibitory) if the earliest statistically significant deviation of the response after sniff onset for the highest odor concentration was an increase (decrease) in spike rate or, if no single bin was statistically significant, and mean firing rate increased (decreased) following odor onset (Fig. 1*A*). Sharp responses were defined using previously established criteria (Shusterman et al., 2011).

#### Quantifying response parameters for individual unit–odor pairs

To examine how responses of individual unit–odor pairs changed with odor concentration and adaptation, we constructed peri-sniff time histogram (PSTH) traces for different odor concentrations and for different sniffs following odor onset. We filtered the response using a 10 ms sliding boxcar window with a 1 ms step. For a given sniff, we defined the following parameters: average firing rate (FR; the mean firing rate during the sniff); peak amplitude (A; the peak of the PSTH for the sniff); and peak latency (L; the time, relative to sniff onset, of the PSTH peak. Figure 1*C* plots the distribution of latency and amplitude on the first sniff for all significant responses.

**Figure 2.**
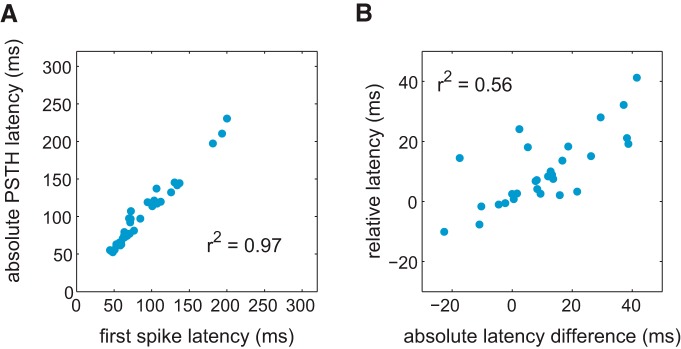
***A***, Latency of the first spike estimated using distributions of interspike intervals (Shusterman et al., 2011) for responses identified as sharp pooled across all odors and concentrations versus the latency of the peak PSTH for the same response. ***B***, Difference in absolute PSTH latency between sharp responses to high and 3× lower concentrations versus the relative latency estimated using cross-correlation (see Materials and Methods).

To examine how response timing changed with concentration, we measured the relative latency from the lag in cross-correlation functions between PSTHs for first sniff responses to the high concentration and for the lower concentration$,{\Delta L}_{0.3-1.0}^{1}$. This enabled us to use a common measure for both positive and negative responses, as well as for responses without a well defined peak. For sharp responses, the relative latency was strongly correlated with the difference in peak latency for the two concentrations (Fig. 2). Positive values of ${\Delta L}_{0.3-1.0}^{1}$ correspond to delayed responses at lower concentrations. To examine changes in amplitude, ΔA, and firing rates, ΔFR, we subtracted values for the high concentration from values for the lower concentration to obtain ${\Delta A}_{0.3-1.0}^{1}=A_{0.3}^{1}-A_{1.0}^{1}$ and ${\Delta FR}_{0.3-1.0}^{1}={FR}_{0.3}^{1}-{FR}_{1.0}^{1}$.

For adaptation, we performed similar analyses, but with responses to the high concentration on the seventh sniff replacing responses for the lower concentration on the first sniff. Thus, positive values of ${\Delta L}_{1.0}^{7-1}$ correspond to delayed responses following adaptation. We also computed changes in amplitude and firing rate as follows: ${\Delta A}_{1.0}^{7-1}=A_{1.0}^{7}-A_{1.0}^{1}$ and ${\Delta FR}_{1.0}^{7-1}={FR}_{1.0}^{7}-{FR}_{1.0}^{1}$.

To determine whether response changes following odor dilution were correlated with response changes following adaptation over the population of recorded unit–odor pairs, we computed Spearman cross-correlations among ΔL, ΔA, and ΔFR values obtained for changes in concentration and with adaptation. Calculations were made separately for excitatory, inhibitory, and sharp responses.

#### Population response vectors

To examine patterns of neuronal activity, for every cell *k* ($k=1,.M_{unit}$), at trial *i* (i=1,...N, where *N* = 50-100), sniff *s* (s=-1,1,...7), and time bin *t* (t=1,.T), we defined the response as a number of spikes in a given bin: $S_{s,i}^{k}(t)$. We constructed the following vectors: average firing rate of cell k at sniff s trial i:

$${\overset{¯}{r}}_{s,i}^{k}=\frac{1}{T}\overset{T}{\underset{t=1}{\sum }}S_{s,i}^{k}\left(t\right),$$

the average firing rate across trials:

$${\overset{¯}{R}}_{s}^{k}=\frac{1}{N}\sum_{i=1}^{N}{\overset{¯}{r}}_{s,i}^{k},$$

temporal pattern of cell k at sniff s at trial i (spike count at a given bin minus average firing rate):

$$r_{s,i}^{k}\left(t\right)=S_{s,i}^{k}\left(t\right)-{\overset{¯}{r}}_{s,i}^{k},$$

and trial averaged temporal pattern:

$$R_{s}^{k}\left(t\right)=\frac{1}{N}\sum_{i=1}^{N}r_{s,i}^{k}\left(t\right).$$

#### Principal component analysis

To examine the principal sources of variability in our dataset, we performed principal components analysis (PCA) on population response vectors (PRVs) for cell–odor pairs recorded across three odor concentrations (mean, 49 cell–odor pairs). The firing rate PRV for each concentration and each sniff (total, 21 vectors) consists of firing rates of individual cell–odor pairs ($R_{s}=\left[{\overset{¯}{R}}_{s}^{1},{\overset{¯}{R}}_{s}^{2},...{\overset{¯}{R}}_{s}^{M}\right]$), while temporal PRV is composed of concatenated trial-averaged PSTHs (200 ms per sniff, binned at 10 ms: *T* = 20 time points/cell) for each sniff and each concentration for all cell–odor pairs: $T_{s}=\left[R_{s}^{1}\left(1\right),R_{s}^{1}\left(2\right),...R_{s}^{1}\left(T\right),R_{s}^{2}\left(1\right)...R_{s}^{M}\left(T\right)\right]$. PCA was performed using the *svd.m* function in MATLAB. The first three principal components (PCs) accounted for the bulk of the variance in the responses. Reduced population vectors were created by reconstructing the population vector using only the first three PCs. To visualize changes across vectors, we projected each response onto the first three principal components from the analysis (see Fig. 5).

To examine the robustness of the PCA solution, we split single-trial responses for each unit–odor–concentration combination into 10 nonoverlapping sets and created 10 sets of PRVs from the resulting PSTHs. We then projected these PRVs into the space of the three PCs generated from average PRVs and computed SD ovals within the space of the first and second and the second and third PC (see Fig 6, small markers, shaded ovals).

#### Classifier analysis

To classify the single-trial population vectors for each sniff s, and each concentration, $c\in \left\{0,0.1,0.3,1.0\right\}$, we constructed a Euclidean distance classifier that classified each vector $r_{s,c,i}={\left[r_{s,i}^{1}(1),r_{s,i}^{1}(2),...r_{s,i}^{M}(T)\right]}_{c}$ as belonging to the group with an average population vector $R_{s,c}={\left[R_{s}^{1}(1),R_{s}^{1}(2),...R_{s}^{M}(T)\right]}_{c}$ for a given sniff and concentration: that was closest to it in the full neural response space according to the following:

$$D\left(r_{s,c,i},R_{s,c}\right)={\left[\sum_{k=1}^{M}\sum_{t=1}^{T}{\left(r_{s,c,i}^{k}\left(t\right)-R_{s,c}^{k}(t)\right)}^{2}\right]}^{\frac{1}{2}}.$$

Single-trial population vectors were created by randomly selecting a single-trial response pattern for each unit of a pool of recorded single-trial responses. This procedure was repeated 250 times for different single-trial population vectors. The selected single-trial responses were excluded from trial averaged vectors. Figure 5 shows the classification between trial-averaged and single-trial vectors on the same sniff, as follows: $r_{s,c,i}\to \left\{R_{s,0},R_{s,0.1},R_{s,0.3},R_{s,1.0}\right\}$. Figure 6 shows the classification between single-trial vectors on different sniffs and trial-averaged vectors on the first sniff, as follows: $r_{s,c,i}\to \left\{R_{1,0},R_{1,0.1},R_{1,0.3},R_{1,1.0}\right\}$. The trial-averaged vector for the blank response $R_{s,0}=〈R_{-1,c}〉$ was included in all classifications.

### Human psychophysics

#### Subjects

Subjects were screened using a comprehensive questionnaire to establish that they had normal olfactory function. Volunteers completed three visits to become acquainted with performing computer-controlled olfactory tasks and then four to eight visits on which perceptual data were collected. Three volunteers (two males, one female; age range, 24–31 years) participated in the study. All experiments were approved by the Institutional Review Board.

#### Odor delivery

Experiments were conducted using a custom-built air dilution olfactometer modeled after that of Bodyak and Slotnick (1999). Briefly, the output of an air compressor (Easy Air, Precision Medical) was charcoal filtered (Vacu-Guard 150/Active Carbon, Whatman) and split into three pressure-regulated lines. One of the lines, labeled “clean” carried 20 L/min filtered air directly to the subject. Flow in the other two lines was digitally controlled by two mass flow controllers (Alicat Scientific) that regulated their combined flow to 2 L/min. These connected into upstream and downstream Teflon manifolds of the olfactometer. Air flowing into the upstream manifold could be directed to one of eight vials containing pure odorant by solenoid pinch valves (BioChem Valve, Neptune Research). Odorized air was then combined with clean air in the upstream manifold. Odor concentration could be controlled by changing the ratio of odorized to clean air (odorized air flow, 0-0.3 L/min; clean air flow, 2-1.7 L/min). To minimize the effects of odor absorption, all tubing (1-2 mm inner diameter) after the odor vial was made of Teflon. Air from the olfactometer was combined with the 20 L/min clean air stream using an additional custom Teflon manifold that terminated in a Teflon-coated mask shaped to fit the human nose (Nasal Ranger). The exhaust port of the mask was routed to a pair of mass flow sensors (AWM720P1, Honeywell) that measured inhalations and exhalations (typical peak flow rates, 50 L/min). Stable odor output and fast kinetics of the olfactometer were confirmed frequently using a photoionization detector (mini-PID, Aurora Scientific). The olfactometer (solenoid opening; changes in odor flow rate) was controlled by custom-made circuitry and software powered by a PC running MATLAB (MathWorks) interfacing with an Arduino Mega 1280 microcontroller.

#### Task

Volunteers sat facing a gray computer screen with their nose inside the odor port and hands placed on the number pad of a keyboard. Initiation of a trial was queued by two brief beeps and a message on the computer screen instructing them to prepare for sniffing. Volunteers were then instructed to make a series of inhalations and exhalations queued by tones (2 s duration). The first inhalation in the series had no odor and served to entrain the subjects’ breathing. Subjects then inhaled an adapting odor concentration (60 ml/min saturated vapor delivered in 22 L/min air) for 0-3 inhalations. After the adaptation period, the flow rate of the odor was changed to one of six test values (0, 15, 30, 60, 120, and 300 ml/min). After making one inhalation of the test concentration, subjects were instructed to rate its perceived intensity on a scale of 0-9. Each trial was separated by a 30 s intertrial interval to reduce the effect of trial-to-trial adaptation.

To calibrate volunteers’ perceptual scale, they performed several test runs where they were presented with the full range of odor concentrations used in the study without adaptation. They were asked to assign 9 for the highest concentration and 0 for no odor. The relative ratings of the intermediate concentrations were at the discretion of the volunteers.

All manipulations were repeated for the following two odors: isoamyl acetate and α-pinene. One volunteer did not adapt to α-pinene, possibly due to the lower overall perceived intensity of this odor and was excluded from analysis of that odor. In each session, volunteers performed five repetitions for each stimulus condition used (4 adaptation durations × 6 concentrations = 24 conditions) resulting in 120 trials per session (total duration = 1.5 h). To obtain stable estimates of perceived intensity, subjects repeated the experiment 2-5 times, resulting in *N* = 10-50 intensity ratings for each stimulus condition.

#### Data analysis

Data for each trial consisted of sniffing traces and numerical perceived intensity ratings. For each subject, we pooled perceived intensity estimates across all sessions and took the mean of perceived intensity for each condition. Average perceived intensity across volunteers was then computed as the mean of the average perceived intensity estimates for each volunteer.

We estimated the relationship between perceived intensity and concentration without adaptation by fitting Hill equations of the following form (Chastrette et al., 1998):

$$I=\frac{I_{m}C^{n}}{C_{ip}^{n}+C^{n}}$$

where *I* is the perceived intensity, C is the concentration, *n* is the hill coefficient, $I_{m}$ is the maximum intensity rating, $C_{50}$ is the concentration at the inflection point. The fits were performed independently for each subject.

Effective concentration was calculated independently for each subject by finding the concentration that best matches the perceived intensity of the stimulus after adaptation from the fitted Hill equation.

## Results

Our dataset comprises recordings from putative MTCs (total, 134 units; 47 single units and 87 multiunits) and breathing/sniffing signals from four awake head-fixed mice, passively sampling one of a few presented odors at two or three different concentrations (total, 209 unit–odor pairs; and 548 unit–odor–concentration combinations). Based on our previous work, in order to analyze the odor responses at sniffs of different durations, we applied the sniff-warping technique, by stretching or compressing the temporal intervals corresponding to inhalation and the rest of the sniff cycle to their mean values (Shusterman et al., 2011). We generated sniff-warped traces of activity (see Materials and Methods) for each unit, each odor, and each concentration of the odor stimuli (PSTHs; Fig. 1*A*).

### MTC responses change with concentration

To quantify response changes as a function of odor concentration, we first grouped responses into 339 unit–odor–concentration sets [concentration–response sets (CRSs)]. Each CRS consists of responses to two presented concentrations with a 3× fold concentration difference (two CRSs for each unit–odor pair if three concentrations were presented, and one CRS if two concentrations were presented). The CRSs were divided into initially excitatory responses (86; 25%), initially inhibitory responses (89; 26%; henceforth, excitatory and inhibitory), and sharp responses (29; 9%), a subset of responses that exhibit large rapid changes in firing rate (Shusterman et al., 2011; Fig. 1*A*,*B*; see Materials and Methods). Each CRS was assigned one of the three response types (excitatory, inhibitory, and sharp) based on the response at the highest concentration measured on the first sniff. For each type, we characterized the responses and response changes with concentration by estimating their latencies, amplitudes, and average firing rates (see Materials and Methods).

In contrast to recordings in the anesthetized state, in awake mice the spontaneous MTC firing rate is relatively high (median, 19 Hz; 25-75% interquartile range (IQR), 12-24 Hz), which precludes the estimation of latency by the timing of the first spike in response to a stimulus (Cang and Isaacson, 2003; Margrie and Schaefer, 2003). Thus, we estimated absolute response latency as the timing of the maximum/minimum of PSTH for the excitatory/inhibitory responses.

#### Excitatory responses

Over the population of all presented concentrations of all odors, excitatory responses tiled the sniff cycle: their peak latencies on the first sniff varied from 52 to 270 ms after inhalation onset (Fig.1*C*). The peak amplitudes of the responses (median, 48 Hz; IQR, 33-67 Hz) were not distributed uniformly across the sniff cycle, with responses in the highest quartile (≥67 Hz, *n* = 38 responses) coming earlier (median latency, 90 ms) relative to responses falling into the lowest quartile (≤33 Hz, *n* = 33) 137 ms (*p* < 0.001, Wilcoxon rank sum test for equal medians). Responses in the highest quartile were predominantly sharp (25 of 38), but none of the lowest quartile responses were sharp.

We next examined how the latency, amplitude, and firing rates of responses changed with odor concentration (Fig.1*C*; Materials and Methods). For the population of 86 excitatory CRSs, reducing odor concentration decreased peak amplitudes by 7.8 Hz (median; *p* < 0.001) and decreased net firing rates by 1.2 Hz (median; *p* < 0.001). Responses to lower concentrations were delayed by a relative latency of 2.9 ms (median; *p* < 0.001 Wilcoxon signed rank test for zero median) compared to high-concentration responses. This relative latency shift was estimated from the time shift of the peak of the cross-correlation function between the responses at the two concentrations. This method was used to avoid errors in estimation of differences in PSTH latency and to create a measure that can be used for both excitatory and inhibitory (see below) responses. Latency changes were particularly apparent for sharp responses, which had median delays of 7.5 ms. For sharp responses, direct estimation of the latency change yielded a median of 9.4 ms (IQR, 0.4-19.4 ms; Fig. 2). These latency changes are smaller than previously reported for first spike latency in anesthetized animals (50 ms shift for a 10-fold dilution (Cang and Isaacson, 2003)), but this could be due to different measures of response latency change (relative or absolute latency vs. time of first spike) and different sniffing patterns in awake and anesthetized states.

#### Inhibitory responses

For inhibitory responses, decreasing odor concentration increased the firing rates at the peak of the inhibitory response (*p* < 0.001; median increase, 0.2 Hz) and increased the overall firing rate (*p* < 0.001; median increase, 2.4 Hz). However, for inhibitory responses, decreasing concentration did not significantly alter relative response latency. These results are consistent with previous data showing enhanced responses of inhibitory cells in the olfactory bulb with increased odor concentration, which may account for the greater inhibition at higher concentrations observed here (Cang and Isaacson, 2003).

#### Early and late responses

Early and late odor responses may play different roles in concentration coding because they may be generated by different cell classes (Fukunaga et al., 2012). To test this hypothesis, we divided response distributions into early (<100 ms after inhalation onset) and late (>100 ms after inhalation onset; Fig. 1*D*). Only excitatory responses (but not sharp excitatory) had statistically significant differences between early and late response distributions. For early excitatory responses, the mean latency change was larger than for late responses (7.3 vs 1.5 ms; *p* = 0.01), and the mean firing rate change was smaller (0.51 vs 1.38 Hz).

### Adaptation mimics the effect of decreased concentration on fine temporal responses of MTCs

We next compared changes of MTC responses resulting from adaptation following repeated odor sampling. For this analysis, we created adaptation response sets (ARSs), in which we paired the higher concentration response from each CRS on the first sniff to responses of the same MTC on the seventh sniff of the same concentration. We then analyzed these ARSs in the same manner as the above CRS analysis.

#### Excitatory responses

**Figure 3.**
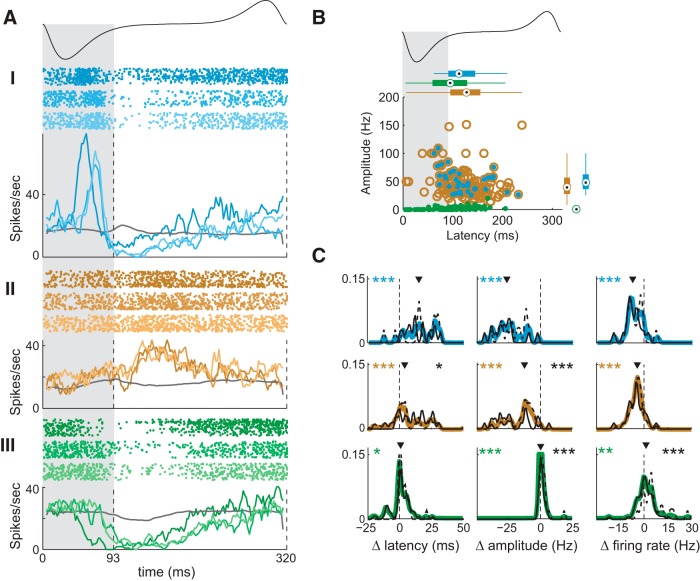
MTC responses change with repeated sampling. ***A***, Sniff-warped raster and PSTH plots of sharp excitatory (I), excitatory (II), and inhibitory (III) responses of single MTCs during the first, fourth, and seventh sniff cycles (shown as color shades). A schematic of the sniff waveform is shown above the plots. Gray shading and vertical dashed lines delineate inhalation period. Gray trace, Activity of the mitral/tufted cell during unodorized sniffs. ***B***, Scatter plot comparing amplitude and latency of excitatory, sharp, and inhibitory responses on the seventh sniff following odor onset. Boxplots show marginal response distributions, as in Figure 1*C*. Color conventions as in Figure 1. ***C***, Colored lines are normalized distributions of changes in latency, amplitude, and firing rate of sharp, excitatory, and inhibitory responses with adaptation (difference between first and seventh sniffs). Black solid and dashed lines are the same distributions for early and late responses. Notations are same as in Figure 1*D*.

Adaptation significantly reduced the amplitude of excitatory responses and increased the relative response latency in a manner similar to a decrease in concentration (Fig. 3). For sharp responses, adaptation decreased the amplitude of peak responses by 12.6 Hz (median; *p* < 0.001), which was associated with a significant reduction in overall firing rates by 4.2 Hz (median; *p* < 0.001). Adaptation also delayed excitatory responses by 4.1 ms (median; *p* < 0.001). Again, latency changes were most pronounced for sharp responses, with median delays of 15.3 ms estimated using cross-correlation and 23.9 ms by direct comparison of latencies.

**Figure 4.**
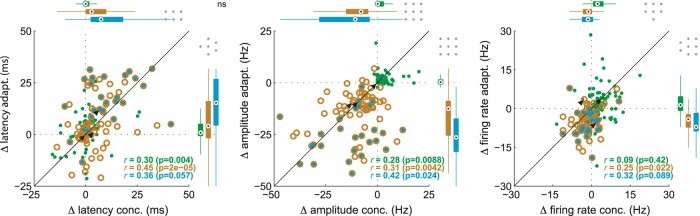
Correlated changes in response features for concentration and adaptation. From left to right, plots show changes in the latency, amplitude, and mean firing rate. Points are individual response sets. Response types are indicated by color as in Figure 1. Box plots show distributions of response changes across cells for concentration and adaptation. Conventions are as in Figure 1. Reported *r* values are Spearman correlation coefficients computed independently for the three response types. Black arrows mark positions of the three example cells in Figures 1 and 2.

Importantly, excitatory response changes induced by adaptation were correlated to changes observed with odor dilution (Fig. 4). We found a significant correlation between the relative latency of (ρ = 0.45, *p* < 0.001) and changes in the amplitude of (ρ = 0.31, *p* = 0.004) odor responses, as well as the average firing rate (ρ = 0.25, *p* = 0.022).

#### Inhibitory responses

For inhibitory responses, adaptation increased the peak firing rate by 0.25 Hz (median; *p* < 0.001) and the overall firing rate by 1.3 Hz (median; *p* = 0.002). Adaptation also tended to delay inhibitory responses by 0.8 ms (median; *p* = 0.018). For inhibitory responses, changes in response timing and amplitude, but not spike rate, were significantly correlated between adaptation and concentration (Fig. 4).

#### Early and late responses

As for concentration dependencies, we compared changes in response adaptation for early and late responses (defined above; Fig. 3*C*). For excitatory responses (but not sharp excitatory), there were significant differences in the adaptation-induced mean response latency change (11.5 vs 2.6 ms, early vs late; *p* = 0.04) and in the mean amplitude change (25.6 vs 11.4 Hz; *p* <0.001). For inhibitory responses, early and late responses differed by the change in amplitude (0.0 vs 2.2 Hz; *p* < 0.001) and firing rate (−0.7 vs −4.7 Hz; *p* < 0.001). No significant differences were observed between early and late sharp responses.

### Total spike count unlikely to explain intensity coding

**Figure 5.**
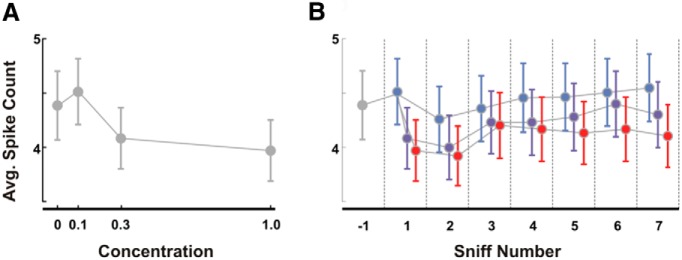
Spike count unlikely to code odor intensity. ***A***, Average number of spikes observed on a single sniff for each unit as a function of odor concentration. ***B***, Average number of spikes per sniff per cell observed on each sniff for the three tested concentrations and baseline (gray, baseline; light blue, 0.1; dark blue, 0.3; red, 1.0). Error bars indicate the SD across trials.

Whereas the temporal activity patterns changed in suggestively similar ways with odor dilution and adaptation, similarity in total spike count (a gross measure of neural activity) was much less compelling. We first counted the total number of spikes in the first sniff observed for different concentrations across all units on a single trial. While some units either increased or decreased their spike counts with concentration, there was little change in total spike count over the full population. Increasing odor concentration tended to decrease the average net spike count: 4.4 ± 0.3 spikes/sniff cycle preodor to 3.9 ± 0.3 spikes for the highest concentration, but this change was not statistically reliable (*p* = 0.09; Fig. 5*A*). Adaptation increased spike count, but again not reliably (Fig. 5*B*). Thus, it is doubtful that the total level of activity is a reliable code for odor intensity (Chalansonnet and Chaput, 1998; Stopfer et al., 2003).

### Adaptation and concentration move population response vectors along a common trajectory in PC space

Odor intensity is likely encoded by the spatiotemporal pattern of activity across many cells in the olfactory bulb (Stopfer et al., 2003; Bathellier et al., 2008). Prior studies (Cury and Uchida, 2010) have suggested that the temporal pattern of MTC activity is consistent with odor-based perceptual decisions. We reasoned that, to be consistent with perception, changes in response patterns from the first sniff to subsequent sniffs should resemble changes observed with odor dilution. To examine how intensity is represented by the temporal activity profile in our population of MTCs, we combined our population of unit–odor pairs into PRVs by concatenating the recorded unit responses. We made separate PRVs for different concentrations and different sniff numbers. To track population response trajectories along a larger concentration range, we used only sessions where odors were presented at the following three different concentrations: 0.1, 0.3, and 1.0 relative to maximal concentration (67 concentration response sets, one set for each unit–odor pair; see Materials and Methods). Thus, we have the following 22 different PRVs: 21 vectors for three concentrations and seven consecutive sniffs, and 1 vector for nonodorized sniff. Each coordinate of these vectors is a deviation of the single-trial spike rate from the average spike rate across sniff for 1 of 67 unit–odor pairs and 1 of 20 time bins during a sniff cycle (total 67 × 20 = 1340 coordinates). We then examined how PRVs change with concentration and with adaptation.

**Figure 6.**
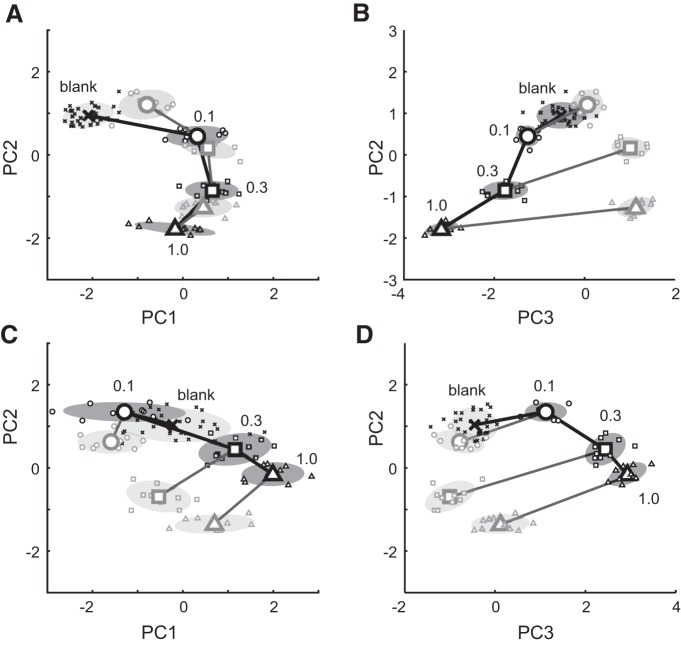
Principal component analysis of the population vector changes with concentration and adaptation. ***A***, ***B***, The full temporal population vectors plotted in the space of the first and second (***A***) and second and third (***B***) principal components. Large symbols, Average PC projection of all first sniffs (black) and seventh sniffs (gray); small symbols, projection of 10 independent subsets of the full dataset (shaded ovals, SD). Blank is cross symbol; concentration 0.1, 0.3, and 1.0, respectively, are circles, squares, and triangles. Black lines connect first sniffs of different concentrations. Gray lines connect first and seventh sniffs of the same concentration. Numbers denote the presented concentrations. ***C***, ***D***, Same as ***A*** and ***B***, but for average firing rate population vector.

To identify the most meaningful dimensions of the response patterns across concentration and sniff number, we reduced the dimensionality of these 22 1340-dimensional vectors using PCA. We visualized responses on each sniff and concentration by plotting the responses in the space of the first three PCs, which accounted for 70% of the total variance (Fig. 6*A*,*B*).

Changes in PRVs with concentration and adaptation were consistent with a representation of odor intensity. PRVs moved smoothly with concentration, creating a curved trajectory away from baseline before odor responses in PC space. Both concentration and adaptation moved PRVs along roughly the same trajectory in the space of the first two PCs (Fig. 6*A*). In this way, responses after adaptation aligned with responses for lower concentrations on the first sniff.

Interestingly, after adaptation the distance between PRVs for different concentrations became smaller, while response variability remained similar (Fig. 6*A*). This suggests that individual concentrations should be more difficult to identify following adaptation.

The consistency with intensity was not observed for population responses composed only of the average firing rates. (Fig. 6*C*,*D*). We performed PCA for response vectors where each coordinate was the average firing rate over a sniff cycle for 1 of 67 unit–odor pairs. Increasing concentration moved these vectors away from baseline in PC space. Adaptation moved firing rate PRVs in a direction different from the concentration decrease. Thus, although both concentration and adaptation changed the pattern of firing rates, their effects on the response were not consistent and therefore not obviously related to intensity coding.

Visual inspection of PCA results provides qualitative intuition for the following two hypotheses: it predicts that (1) adaptation increases errors in concentration discrimination, and (2) adaptation decreases encoded odor concentration. To test these hypotheses quantitatively, we performed single-trial discriminant analysis of MTC population responses. In addition, we examine the perceptual implications of the above hypotheses by measuring the effect adaptation and concentration change on human intensity perception.

### Single-trial discriminant analysis

Animals make decisions based on odor information available in a single trial. We estimated how much information is carried by the spatiotemporal pattern of MTC activity in single trial in the first and subsequent sniffs using discriminant analysis (see Materials and Methods). As for PCA, we used sessions in which three different concentrations were presented (67 unit–odor pairs). We considered all unit–odor pairs independent and equivalent to different cell responses to one odor.

#### Adaptation increases errors in identifying odor concentrations

**Figure 7.**
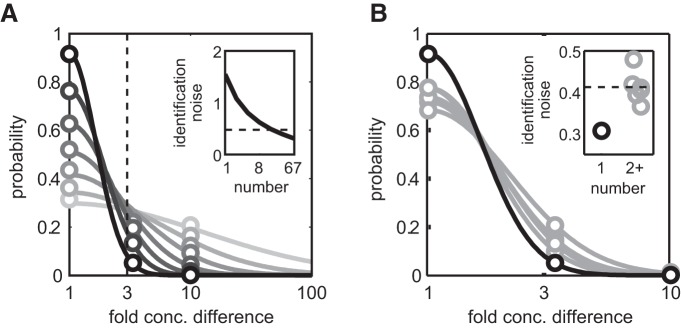
Adaptation increases concentration identification error. ***A***, Results of classification analysis for concentration discrimination between four levels (0.0, 0.1, 0.3, 1.0): average probability of classification (empty circles) of temporal patterns of MTCs at the first sniff as a function of concentration mismatch between actual concentration and classified concentration (1 corresponds to correct classification, 3(10) is the classification mismatch for 1(2) step threefold concentration differences) for different numbers of cells (shading from lightest to darkest corresponds to 1, 2, 4, 8, 16, 32, and 67 cells). Solid lines are Gaussian fits of classification probability: $p=p_{1}exp\left(-{\left(\Delta {log}_{10}\left(C\right)\right)}^{2}/\sigma^{2}\right)$, where *p*1 is a probability of correct classification, and σ is the concentration classification error in log10 units. Inset, Concentration classification error as a function of number of cells included in classification. Vertical dashed line: threefold concentration difference. ***B***, Classification performance for all 67 cells for different sniffs following odor onset (black, sniff 1; gray, sniffs 2-7). Inset: concentration classification error for sniff 1 (black) vs later sniffs (gray). Dashed line: median for sniffs 2+.

On the first sniff, a single responsive MTC can, on average, identify the presented concentration level (0.0, 0.1, 0.3, or 1.0) at 31% accuracy, which is slightly higher than chance (25%). However, identification accuracy quickly increased as more units were included in the analysis, reaching 92% for the maximal number of recorded unit–odor pairs (*n* = 67; Fig. 7*A*). With a single unit, the average probabilities of misidentifying a given concentration as 3× or even 10× different were nearly equal. Increasing the number of units in the analysis abolished errors to 10×, and the analysis using a maximal number of units nearly abolished errors to 3× concentration differences. This means that most classification errors are made in adjacent concentrations in a manner consistent with a graded code for concentration. To capture this effect, we estimated the concentration identification noise, σ, as the width of a Gaussian fit to our classification results as a function of concentration difference (in log units): $\Delta log\left(C\right)$: $p=p_{1}exp\left(-{\left(\Delta {log}_{10}\left(C\right)\right)}^{2}/\sigma^{2}\right)$ (Fig. 7*A*). For a single MTC, the average concentration identification noise was equal to 1.63 (corresponding to a 43-fold concentration difference), decreasing to just 0.3 log units (2-fold) for our full population of responses (Fig. 7*A*, inset).

We next examined classifier performance after adaptation using our full population of responses. Correct identification performance decreased from 92% on the first sniff to 68-78% on subsequent sniffs (Fig. 7*B*; while identification noise increased from 0.3 to 0.4 log units (2.6-fold concentration difference; *t* test, *p* = 0.048; Fig. 7*B*, inset). As for the first sniff, misidentification errors on later sniffs occurred between similar concentrations.

Thus, although concentration information was still largely intact after adaptation, odor concentrations were harder to distinguish, as suggested earlier by PCA.

#### Adaptation reduces coded odor concentration

**Figure 8.**
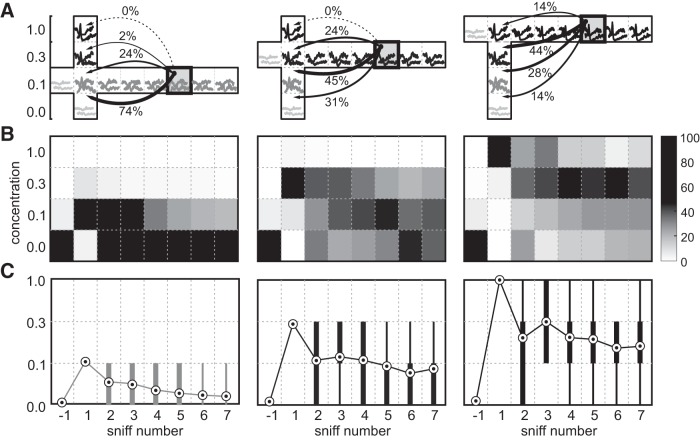
Adaptation decreases the encoded odor concentration. Single-trial responses were classified based on their Euclidean distance to the average responses to the three concentrations presented on the first sniff and the average blank response. ***A***, Schematics of the classification process for three concentrations (left, 0.1; middle, 0.3; right, 1.0). Responses on a given sniff and concentration (examples are shown in boxes) are classified against responses on the first sniff. The arrows from sniff 5 (shaded box) illustrate match probabilities between this sniff and responses on the first sniff. ***B***, For each concentration (left to right), grayscale plots show the classifier match probability (see bar on right) for responses on a given sniff (*x*-axis) with the average concentration responses on the first sniff (*y*-axis). ***C***, Equivalent concentration for each sniff calculated as the average match probability weighted by concentration (circles), and distributions of classification results: thin line is the 10-90% interval; and thick lines are the 25-75% interval.

Our PCA analysis suggested that responses to odors following adaptation should become more similar to lower odor concentrations. Using discriminant analysis, we classified responses on consecutive sniffs for a given concentration based on their similarity to the average responses on the first sniff at different concentrations (Fig. 8). As predicted, responses on later sniffs were preferentially matched to lower concentrations, but rarely to higher concentrations. For each presented concentration on each sniff, we estimated the “effective” concentration as the best matching concentration on the first sniff. To do this, we computed the sum of the presented concentrations weighted by the match probability between a given sniff concentration response and concentration responses on the first sniff (0.1, 0.3, 1.0) and also baseline (concentration, 0). This measure of effective concentration decreased abruptly after the first sniff and quickly reached a steady state corresponding to a 3- to 10-fold lower concentration (Fig. 8).

### Adaptation reduces perceived intensity ratings, increasing rating noise

**Figure 9.**
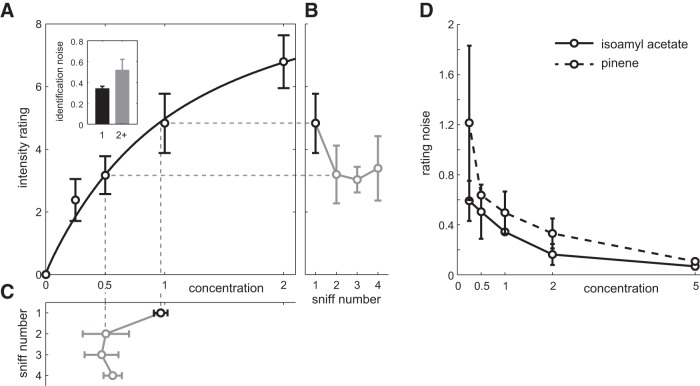
Effect of adaptation on perceived odor intensity. ***A***, Average intensity ratings for different concentrations of the odor pinene obtained on the first sniff (black) and after adaptation (gray). Curve denotes average Hill equation fit between concentration and perceived intensity. Concentration has been normalized such that concentration 1 corresponds to 60 ml/min saturated vapor diluted in a typical 2 s inhalation and a peak flow rate of 50 L/min (minimum 0.12% saturated vapor). Inset, Rating noise (rating SD/mean). ***B***, Perceived intensity of the odor stimulus with concentration 1 across sniffs from a constant odor source. ***C***, Equivalent concentration computed as the concentration with the same intensity rating on the first sniff extrapolated from the Hill equation fit for individual subjects (schematized by dashed gray lines). Error bars indicate the SD across subjects included in the analysis. ***D***, Rating noise as a function of presented odor concentration for pinene (dashed) and isoamyl acetate (solid).

To further develop our understanding of the relationship between adaptation and concentration changes, we performed psychophysical experiments with human subjects. We asked human volunteers to rate the perceived intensity of odors across sniffs. We measured the perceived intensity of odors across several consecutive inhalations in three subjects (Fig. 9). Volunteers were asked to rate a panel of odor concentrations presented either on the first sniff or after several sniffs of an adapting concentration. In general agreement with prior work (Moncrieff, 1957; Engen, 1964; Cain, 1970; Stone et al., 1972), average intensity ratings followed a nonlinear relationship with odor concentration that is well described by the Hill equation (Chastrette et al., 1998; odor isoamyl acetate; Fig. 9*A*). We quantified the trial-to-trial variability of perceived intensity ratings as a function of the presented concentration. To do this, we computed rating noise as the ratio of the SD of intensity ratings relative to their mean (Fig 9*D*). Rating noise decreased significantly with odor concentration. For isoamyl acetate, rating noise decreased on average across subjects from (mean ± SD) 0.59 ± 0.16 at the lowest concentration to 0.07 ± 0.02 at the highest.

Prolonged exposure to a constant odor source decreased mean intensity ratings (33 ± 0.02% decrease for isoamyl acetate). Converting from intensity to concentration units using the fitted Hill equation showed that these lower ratings corresponded to a roughly twofold dilution of the odor (Materials and Methods; Fig. 9*C*). Whereas the mean of the perceived intensity ratings decreased with adaptation, the rating noise increased from 0.34 ± 0.02 to 0.52 ± 0.10. We observed similar effects across different odors (see Materials and Methods). These results of human psychophysics experiments are consistent with observations made from MTC responses: namely, that the effective concentration of odors quickly decreases after the first sniff with an associated increase in identification noise.

## Discussion

Here we investigated the neural representation of odor intensity in the olfactory bulb of awake mice. We find that MTC odor responses change with decreasing concentration, similar to that with repeated sampling of a constant odor source. We used our recorded population of MTCs to decode odor concentration using classifier analysis. On the first sniff, MTCs reliably identified the presented odor concentration to within a factor of 2, but identification noise increased on later sniffs. Using first sniff responses to classify concentrations on later sniffs resulted in poor performance because responses on later sniffs were systematically misclassified as lower concentrations. These neural results are consistent with changes in perceived odor intensity across sniffs reported by human volunteers. Repeated sampling of a constant odor source caused a decline of perceived intensity ratings and an associated increase in rating noise. Our data suggest that responses of neurons in the olfactory bulb are consistent with the perceptual feature of odor intensity.

### A representation of odor intensity on each sniff

Rodents and humans can make decisions based on a single sniff of odor (Laing, 1986; Uchida and Mainen, 2003; Kepecs et al., 2006). This implies that animals’ olfactory percept is regenerated, or at least refreshed, on each sniff by the incoming pattern of MTC activity. Thus, a constant odor source does not present a static input into the olfactory system but is converted, by sniffing, into discrete samples. Consistent with prior studies, we find that the pattern of MTC activity on each individual sniff carries a robust code for odor concentration (Gross-Isseroff and Lancet, 1988; Chalansonnet and Chaput, 1998; Bathellier et al., 2008; Zhou and Belluscio, 2012; Patterson et al., 2013). This code has been shown to change significantly with repeated sampling, reducing the ability of classifiers to correctly identify the presented concentration when using the first sniff as a response template (Bathellier et al., 2008; Patterson et al., 2013).

We find that reduced classification accuracy observed across sniffs is not due to random drifts in the neural response over time, but rather to systematic decreases in the coded odor concentration. Over the population of recorded MTCs, the peak response amplitude, response latency, and firing rate changed from the first to subsequent sniffs. Further, these response changes were significantly correlated with how responses change with odor dilution. Principal component analysis of MTC population responses illustrated that concentration and adaptation have similar trajectories in PC space, with responses after adaptation becoming systematically more similar to responses to lower concentrations. Finally, whereas our classifier analysis quantitatively confirmed prior findings of reduced classification accuracy between sniffs, this effect was dominated by classification errors to 3× to 10× lower odor concentrations. These data suggest that each sniff of a constant odor source generates a new odor percept, with perceived intensity falling immediately after the first sniff.

Though most of the variability in MTC responses could be attributed to changes in odor intensity, responses after adaptation were also significantly different from any of the responses on the first sniff. These differences were clearly captured by the third principal component in our PC analysis (Fig. 4*B*), suggesting that perceptual properties other than intensity (e.g., odor quality) change in different ways with adaptation compared with concentration.

### Early and late responses do not show differences in intensity coding

Prior work proposed that responses of tufted and mitral cells have different concentration dependence (Fukunaga et al., 2012). In the anesthetized state, odor responses of tufted cells peaked early after inhalation onset and had very small latency shifts with concentration, whereas the mitral cells responded late and had a much greater shift in latency with concentration. Our recordings in the awake state did not show a similar relationship between early and late responses, and their latency shifts with concentration, and do not allow us to differentiate cell types. Therefore, our data cannot determine whether mitral and tufted cells participate differently in intensity coding.

### Possible mechanisms for similar MTC responses changes for adaptation and concentration decrease

At the receptor level, adaptation and concentration have different effects. Increasing odor concentration usually leads to recruitment of a larger number of olfactory receptor neurons (ORNs; Bozza et al., 2004; Grosmaitre et al., 2006). Prior work (Duchamp-Viret et al., 2000) shows that increasing odor concentration can increase ORN spike counts and reduce response latency. Strong peripheral adaptation at the level of ORNs was reported for high concentrations of odor, while responses to lower odor concentration were mainly unaltered (Lecoq et al., 2009). This effect may explain the change in perception of odor identity for high concentrations and may be unrelated to the perception of odor intensity. Investigations in humans attempted to relate receptor activity to perception of intensity using electro-olfactograms (EOGs), a measurement reflecting mass action of olfactory receptors. Despite good correlation between changes of EOG amplitudes with concentration and changes in perceived intensity with concentration (Lapid et al., 2011), the two measures were dissociated by adaptation. While perceived intensity was greatly reduced by repeated odor sampling, EOG amplitudes remained virtually unchanged (Hummel et al., 1996). This casts doubt on receptor-based explanations of perceived intensity based on pooled receptor response magnitudes. Alternatively, feedback within olfactory bulb or from higher olfactory areas may alter odor representation after the first sniff of odor (Patterson et al., 2013), which could mimic the concentration decrease. In addition, granule cells, which show little response modulation by respiration in the awake state (Cazakoff et al., 2014), may be a good candidate for suppressing and delaying responses across sniffs.

### Responses following adaptation are compressed along the intensity axis

Our finding of a 3× to 10× drop in the odor concentration coded by populations of MTCs in mice is strikingly similar to the decrease in perceived odor intensity measured in rats (Wojcik and Sirotin, 2014) and humans (Engen, 1964; Ekman et al., 1967; Cain, 1970; Pryor et al., 1970; Steinmetz et al., 1970; Wojcik and Sirotin, 2014; Cain and Engen, 1969). Wojcik and Sirotin (2014) found that the relative perceived intensity of an odor following adaptation falls by a factor 3× to 10× following a brief 300 ms exposure depending on odor type. This adaptation period corresponds to roughly two sniffs. Even a single sniff of odor in human volunteers was sufficient to decrease the perceived odor intensity by a factor of 2×. Thus, decreases in perceived intensity are generally consistent with changes in concentration coding at the level of MTCs.

In addition to a decrease in the coded odor concentration, classifier analysis of MTC responses showed that later sniffs were associated with a greater number of errors (identification noise) compared with the first sniff. There are two possible explanations of this result: an increase in the variability of intensity responses on later sniffs; or constant variability but with adapted responses closer together along the intensity axis. Our PCA showed that responses after adaptation moved closer to lower concentration responses, but were not significantly more variable. Perceptual data from human volunteers showed that the across-trial variability in intensity ratings was constant across the full range of mean rated intensity. This caused the intensity rating noise to increase with decreasing stimulus intensity. Decreases in intensity with adaptation were also accompanied by increased rating noise. This finding is consistent with responses following adaptation being compressed along the intensity axis while the noise in the represented concentration remains fixed.

### Which features of the neural response carry intensity information?

Although our results demonstrate that neural responses of MTCs are broadly consistent with a representation of odor intensity, all examined features of MTC activity changed in similar ways with concentration and adaptation. Of the examined features, the relationship was weakest for changes in mean firing rate across the sniff cycle, and PCA of the firing rate pattern across MTCs did not show any systematic links between response changes with concentration and adaptation. However, because any of the examined neural features can likely be read out behaviorally and may influence perception (Smear et al., 2013), it is difficult to assign any one a causal role. Prior studies (Koulakov et al., 2007; Schaefer and Margrie, 2007; Zhou and Belluscio, 2012) have suggested a number of ways in which odor intensity may be represented in the olfactory bulb. In the future, these plausible theories can be tested using targeted trial-by-trial perturbations of neural activity combined with perception in the same animals.

### Comparing olfactory perception across species

Despite dramatic phylogenetic differences, general principles of olfactory structure and coding appear conserved among mammals, fish, and insects. In all species, axons from olfactory sensory neurons are pooled into glomeruli, where they synapse onto principal neurons (MTCs in mammals and fish, and principal neurons in insects) embedded in an inhibitory network. In all systems examined, these principal neurons respond to odors with spatiotemporal activity patterns (Laurent, 2002) that refine the odor representation before sending it to higher brain areas (Mori, 1999; Friedrich and Laurent, 2001). Because of such structural and functional homology across phyla, it is likely that neural mechanisms of odor coding are also conserved.

In this study, we compared perceptual adaptation in humans with MTC odor responses in awake mice. Despite significant differences in sampling behavior (0.25 Hz sniffing in humans; 3 Hz sniffing in mice), the magnitude and even the fast kinetics of adaptation appear comparable across species (Smith et al., 2010; Wojcik and Sirotin, 2014). We monitored neural data across seven sniffs of odor by mice, which correspond to >2 s of odor exposure, similar to one human inhalation. Odor adaptation in olfactory sensory neurons can be long lasting (Zufall and Leinders-Zufall, 2000; Patterson et al., 2013). Thus, the drop in perceived intensity of on the second inhalation of odor in our volunteers may indeed be mediated by neural mechanisms similar in quality to the observed changes in mouse MTC responses. We suggest that the insight gained from measuring human perception can serve as a synergistic tool for understanding neural representations and coding in olfaction (Zelano and Sobel, 2005), just as these comparisons have been useful in understanding other sensory modalities (Mountcastle et al., 1963; Johnson et al., 2002).

Relating olfactory perception to neural responses can help elucidate how and where odor percepts are represented in the olfactory system. In other systems, this approach led to significant insight into the representation of perceptual features (Mountcastle et al., 1963; Britten et al., 1996; Hernández et al., 2000; Yoshioka et al., 2001; Liu et al., 2013). One idea that has been put forth is that any candidate neural code for a specific perceptual feature must show consistency with perception (Johnson et al., 2002). Here we demonstrate that the sniff-triggered temporal pattern of neural responses in the olfactory bulb changes in a manner similar to that of odor dilution and adaptation, showing qualitative consistency with the perceptual phenomenon of adaptation. It may be useful to apply this approach to investigating links between other perceptual and neurophysiological phenomena, such as olfactory afterimages (Patterson et al., 2013) or masking (Cain, 1975).

## Synthesis

The decision was a result of the Reviewing Editor Upinder Bhalla and the peer reviewers coming together and discussing their recommendations until a consensus was reached. A fact-based synthesis statement explaining their decision and outlining what is needed to prepare a revision is listed below. The following reviewers agreed to reveal their identity: Lowe Graeme, Venkatesh Murthy

The authors analyze how firing patterns in mitral/tufted cells convey odor intensity, including adaptation effects. The study provides a strong foundation for understanding neural intensity coding through a combination of physiology and behavior

The reviewers both feel that the study substantially advances the field. It is methodologically strong, and the results are clear. While the intensity code cannot be definitively assigned through the current data, the study still represents a big step.

While it would be desirable to precisely identify Mitral vs. Tufted cells, the reviewers agree that with the present technique this is not feasible to do this definitively. Instead the authors are encouraged to classify units by latency, and use this to compare putative groupings of cell types. The authors are also encouraged to discuss a possible mechanistic basis for the similarity of MTC responses in the cases of adaptation and concentration.

Reviewer 1.

This study examines in depth how spike firing patterns of mitral/ tufted cells (MTCs) in mouse olfactory bulb shift with odor adaptation over time, and tests if these changes match the changes in responses seen with decreasing odor concentration. Matching is a necessary (but not sufficient) condition for a neural code of intensity, because intensity percept similarly decreases with both adaptation &amp; concentration. The authors report that indeed MTC spike patterns in adapted states resemble more closely MTC responses to concentrations lower than the adapting one. This is significant because quantitative analysis of MTC adaptation has not been done in this level of detail. It is an impressive technical achievement in awake behaving animals. The adaptation of MTC properties including noise, were also matched to human perceptual data on a sniff-by-sniff basis. The results show that an intensity code can reside in MTC spiking patterns, but it remains unclear what aspects of the patterns ae used to code for intensity. Both response amplitudes and latency showed adaptation-vs-concentration scalings, so neither were ruled out; the average firing rate or spikes per sniff appears least likely to code for intensity.

One limitation here is not knowing what portion of recorded units are mitral vs tufted cells. Recent studies show that tufted cells differ from mitral cells in a number of properties, e.g. higher excitability and peak firing rates, less regular firing patterns, locking to different sniff phase, shorter and more concentration-invariant latencies, weaker lateral inhibition, different cortical projections, etc. The two cell types might contribute differently to intensity coding, which would be obscured by lumping them together. Although the blind in-vivo recording method precludes positive identification and segregation of cell types, might it be possible to investigate this by comparing adaptation-vs-concentration scalings between subsets of recorded units categorized as either 'tufted-like' vs 'mitral-like' based on certain criteria? For example, if shorter latency units were analyzed collectively as a putative 'tufted-like' group, would they show better ot worse adaptation-vs-concentration scaling than the other units with longer latencies (putative 'mitral')? The authors have compared 3 subclasses: 'sharp excitatory' vs. 'excitatory' vs. 'inhibitory', but these classifications may not correlate with tufted vs. mitral types.

Perceived odor intensity can vary not only with concentration and adaptation, but maybe also with odorant chemical structure or physicochemical properties. A diverse panel of 17 different odorants was tested. How can this potential variable be factored into the analysis?

The authors did not discuss any mechanistic basis for their findings, i.e what underlies the observed adaptation-vs-concentration matching of MTCs responses? Given that olfactory sensory neurons (OSN) in the periphery adapt strongly to odor stimuli, i.e. OSN adapted responses to high concentration tend to resemble OSN responses to low concentration, it is conceivable that MTC properties described here simply reflect the similar adaptive properties of the OSN inputs to the bulb. Lecoq &amp; al (2009) showed that sniff-wise adaptation could be attributed primarily to OSN presynaptic adaptation, rather than postsynaptic processing by bulb circuits.

Reviewer 2.

In this manuscript, the authors describe a thorough neurophysiological and behavioral analysis of intensity perception in olfaction. The authors first perform extracellular spike recordings in the olfactory bulb of behaving mice to show that principal neurons represent odor concentration most robustly using some sort of a latency code - such that the early spikes (peak response, for example) provides much clearer information for any classifier than the average response over a longer period (sniff duration). The authors also show that this code is also evident when considering adaptation over multiple sniffs (ie, with extended presence of the odorant). They perform exciting correlation between behavior of mice and humans, and offer strong evidence that intensity coding is most likely through a temporal code (I suspect they really mean a latency code, where the earliest responses are the most informative). The remarkable rigor of the experiments, the clarity of the methods and the clear-cut results will ensure that this work will be cited and followed up for years to come. I can see that this may be the sort of paper that prompts many a reviewer to sharpen their knives and point to numerous earlier papers that purport to make similar points. I feel, however, the rigor with which the points are made is unparalleled and the work should be made public as soon as possible, and the community can weigh in (I am betting it will be very positive).

I have no objection for this paper to be published, but have some technical comments.

Page 5/6: The sniff measurements and odor delivery calibrations are excellent calibration, as are the careful techniques for timing. This is an important and unique part of the paper that distinguishes it from other previous work.

Page 6, line 6: the clause about directing to exhaust is confusing- I think they mean that the odor is directed to exhaust during the waiting period.

Page 7, line 8: ...(200 and 500ms)... I think the range between these two numbers is intended. If so, say between 200ms and 500ms

Page 7, bottom lines: figures are cited in methods and are cited out of order.... Not sure what the rules are for eNeuro.

Page 9, line 8: "concatenation " is the intended word, I think.

Page 12, line 1 of Results: multi units seem problematic since subunits may do opposite things. I am not sure if the authors ensured that only using single units gave essentially the same results.

Page 14, 7 or 8 lines from bottom: this difference between delays measured using two different methods seems quite significant (15.3 vs. 23.9ms) - does this matter?

Page 15 top and elsewhere: The frequent reference to Cang and Isaacson is generous, but seems unnecessary given that those experiments were all in anesthetized animals, and Isaacson himself has shown that life is quite different when awake. Fine with me, but I'd support fewer allusions to that paper.

Page 15: total spike count section. Seems like a hold out from a previous submission, but the reference should be to Fig 5 and not Fig S5.

Figures: for future reference, I would prefer if the figures had figure legends embedded in them - this is after all the digital age, and we don't need to scroll back and forth for matching figures with text.

## References

[B1] Abraham NM, Spors H, Carleton A, Margrie TW, Kuner T, Schaefer AT (2004) Maintaining accuracy at the expense of speed: stimulus similarity defines odor discrimination time in mice. Neuron 44:865–876. 10.1016/j.neuron.2004.11.017 15572116

[B2] Anderson AK, Christoff K, Stappen I, Panitz D, Ghahremani DG, Glover G, Gabrieli JDE, Sobel N (2003) Dissociated neural representations of intensity and valence in human olfaction. Nat Neurosci 6:196–202. 10.1038/nn1001 12536208

[B3] Bathellier B, Buhl DL, Accolla R, Carleton A (2008) Dynamic ensemble odor coding in the mammalian olfactory bulb: sensory information at different timescales. Neuron 57:586–598. 10.1016/j.neuron.2008.02.011 18304487

[B4] Beck A, Kruger L, Calabresi P (1954) Observations on olfactory intensity. I. Training procedure, methods, and data for two aliphatic homologous series. Ann N Y Acad Sci 58:225–238. 13139350 10.1111/j.1749-6632.1954.tb54856.x

[B5] Bodyak N, Slotnick B (1999) Performance of mice in an automated olfactometer: odor detection, discrimination and odor memory. Chem Senses 24:637–645. 10587496 10.1093/chemse/24.6.637

[B6] Bozza T, McGann JP, Mombaerts P, Wachowiak M (2004) In vivo imaging of neuronal activity by targeted expression of a genetically encoded probe in the mouse. Neuron 42:9–21. 15066261 10.1016/s0896-6273(04)00144-8

[B7] Britten KH, Newsome WT, Shadlen MN, Celebrini S, Movshon JA (1996) A relationship between behavioral choice and the visual responses of neurons in macaque MT. Vis Neurosci 13:87–100. 8730992 10.1017/s095252380000715x

[B8] Britten KH, Shadlen MN, Newsome WT, Movshon JA (1992) The analysis of visual motion: a comparison of neuronal and psychophysical performance. J Neurosci 12:4745–4765. 1464765 10.1523/JNEUROSCI.12-12-04745.1992 PMC6575768

[B9] Cain WS (1969) Odor intensity: differences in the exponent of the psychophysical function. Percept Psychophys 6:349–354. 10.3758/BF03212789

[B10] Cain WS (1970) Odor intensity after self-adaptation and cross-adaptation. Percept Psychophys 7:271–275. 10.3758/BF03210163

[B11] Cain WS (1975) Odor intensity: mixtures and masking. Chem Senses 1:339–352. 10.1093/chemse/1.3.339

[B12] Cain WS, Engen T (1969) Olfactory adaptation and the scaling of odor intensity In: Olfaction and Taste III (Pfaffmann C, ed), pp 127–141. New York: Rockefeller UP.

[B13] Cang J, Isaacson JS (2003) *In vivo* whole-cell recording of odor-evoked synaptic transmission in the rat olfactory bulb. J Neurosci 23:4108–4116. 12764098 10.1523/JNEUROSCI.23-10-04108.2003 PMC6741073

[B14] Cazakoff BN, Lau BYB, Crump KL, Demmer HS, Shea SD (2014) Broadly tuned and respiration-independent inhibition in the olfactory bulb of awake mice. Nat Neurosci 17:569–576. 10.1038/nn.3669 24584050

[B15] Chalansonnet M, Chaput MA (1998) Olfactory bulb output cell temporal response patterns to increasing odor concentrations in freely breathing rats. Chem Senses 23:1–9. 9530964 10.1093/chemse/23.1.1

[B16] Chastrette M, Thomas-Danguin T, Rallet E (1998) Modelling the human olfactory stimulus-response function. Chem Senses 23:181–196. 9589166 10.1093/chemse/23.2.181

[B17] Cury KM, Uchida N (2010) Robust odor coding via inhalation-coupled transient activity in the mammalian olfactory bulb. Neuron 68:570–585. 10.1016/j.neuron.2010.09.040 21040855

[B18] Duchamp-Viret P, Duchamp A, Chaput MA (2000) Peripheral odor coding in the rat and frog: quality and intensity specification. J Neurosci 20:2383–2390. 10704512 10.1523/JNEUROSCI.20-06-02383.2000 PMC6772510

[B19] Edwards PA, Jurs PC (1989) Correlation of odor intensities with structural properties of odorants. Chem Senses 14:281–291. 10.1093/chemse/14.2.281

[B20] Ekman G, Berglund B, Berglund U, Lindvall T (1967) Perceived intensity of odor as a function of time of adaptation. Scand J Psychol 8:177–186. 6079317 10.1111/j.1467-9450.1967.tb01392.x

[B21] Engen T (1964) Psychophysical scaling of odor intensity and quality. Ann N Y Acad Sci 116:504–516. 14220543 10.1111/j.1749-6632.1964.tb45080.x

[B22] Friedrich RW, Laurent G (2001) Dynamic optimization of odor representations by slow temporal patterning of mitral cell activity. Science 291:889–894. 10.1126/science.291.5505.889 11157170

[B23] Fukunaga I, Berning M, Kollo M, Schmaltz A, Schaefer AT (2012) Two distinct channels of olfactory bulb output. Neuron 75:320–329. 10.1016/j.neuron.2012.05.017 22841316

[B24] Grosmaitre X, Vassalli A, Mombaerts P, Shepherd GM, Ma M (2006) Odorant responses of olfactory sensory neurons expressing the odorant receptor MOR23: a patch clamp analysis in gene-targeted mice. Proc Natl Acad Sci U S A 103:1970–1975. 10.1073/pnas.0508491103 16446455 PMC1413638

[B25] Gross-Isseroff R, Lancet D (1988) Concentration-dependent changes of perceived odor quality. Chem Senses 13:191–204. 10.1093/chemse/13.2.191

[B26] Hernández A, Zainos A, Romo R (2000) Neuronal correlates of sensory discrimination in the somatosensory cortex. Proc Natl Acad Sci U S A 97:6191–6196. 10.1073/pnas.120018597 10811922 PMC18580

[B27] Hopfield JJ (1995) Pattern recognition computation using action potential timing for stimulus representation. Nature 376:33–36. 10.1038/376033a0 7596429

[B28] Hummel T, Knecht M, Kobal G (1996) Peripherally obtained electrophysiological responses to olfactory stimulation in man: electro-olfactograms exhibit a smaller degree of desensitization compared with subjective intensity estimates. Brain Res 717:160–164. 8738266 10.1016/0006-8993(96)00094-7

[B29] Johnson KO, Hsiao SS, Yoshioka T (2002) Neural coding and the basic law of psychophysics. Neuroscientist 8:111–121. 11954556 10.1177/107385840200800207 PMC1994651

[B30] Kato HK, Chu MW, Isaacson JS, Komiyama T (2012) Dynamic sensory representations in the olfactory bulb: modulation by wakefulness and experience. Neuron 76:962–975. 10.1016/j.neuron.2012.09.037 23217744 PMC3523713

[B31] Kepecs A, Uchida N, Mainen ZF (2006) The sniff as a unit of olfactory processing. Chem Senses 31:167–179. 10.1093/chemse/bjj016 16339265

[B32] Koulakov A, Gelperin A, Rinberg D (2007) Olfactory coding with all-or-nothing glomeruli. J Neurophysiol 98:3134–3142. 10.1152/jn.00560.2007 17855585

[B33] Laing DG (1986) Identification of single dissimilar odors is achieved by humans with a single sniff. Physiol Behav 37:163–170 3737714 10.1016/0031-9384(86)90400-2

[B34] Lapid H, Shushan S, Plotkin A, Voet H, Roth Y, Hummel T, Schneidman E, Sobel N (2011) Neural activity at the human olfactory epithelium reflects olfactory perception. Nat Neurosci 14:1455–1461. 10.1038/nn.2926 21946326

[B35] Laurent G (2002) Olfactory network dynamics and the coding of multidimensional signals. Nat Rev Neurosci 3:884–895. 10.1038/nrn964 12415296

[B36] Lecoq J, Tiret P, Charpak S (2009) Peripheral adaptation codes for high odor concentration in glomeruli. J Neurosci 29:3067–3072. 10.1523/JNEUROSCI.6187-08.2009 19279243 PMC6666444

[B37] Liu S, Gu Y, Deangelis GC, Angelaki DE (2013) Choice-related activity and correlated noise in subcortical vestibular neurons. Nat Neurosci 16:89-97 10.1038/nn.3267 23178975 PMC3612962

[B38] Margrie TW, Schaefer AT (2003) Theta oscillation coupled spike latencies yield computational vigour in a mammalian sensory system. J Physiol 546:363–374. 12527724 10.1113/jphysiol.2002.031245 PMC2342519

[B39] Marks L (1978 ) The unity of the senses. San Diego, CA: Academic.

[B40] Moncrieff RW (1957) Olfactory adaptation and odor-intensity. Am J Psychol 70:1–20. 13411290

[B41] Mori K (1999) The olfactory bulb: coding and processing of odor molecule information. Science 286:711–715. 10531048 10.1126/science.286.5440.711

[B42] Moskowitz HR, Dravnieks A, Klarman LA (1976) Odor intensity and pleasantness for a diverse set of odorants. Percept Psychophys 19:122–128. 10.3758/BF03204218

[B43] Mountcastle VB, Poggio GF, Werner G (1963) The relation of thalamic cell response to peripheral stimuli varied over an intensive continuum. J Neurophysiol 26:807–834. 14065329 10.1152/jn.1963.26.5.807

[B44] Over R, Mackintosh NJ (1969) Cross-modal transfer of intensity discrimination by rats. Nature 224:918–919. 5352904 10.1038/224918a0

[B45] Patterson MA, Lagier S, Carleton A (2013) Odor representations in the olfactory bulb evolve after the first breath and persist as an odor afterimage. Proc Natl Acad Sci U S A 110:E3340–E3349. 10.1073/pnas.1303873110 23918364 PMC3761593

[B46] Pryor GT, Steinmetz G, Stone H (1970) Changes in absolute detection threshold and in subjective intensity of suprathreshold stimuli during olfactory adaptation and recovery. Perception Psychophys 8:331–335. 10.3758/BF03212603

[B47] Rinberg D (2006) Sparse odor coding in awake behaving mice. J Neurosci 26:8857–8865. 10.1523/JNEUROSCI.0884-06.2006 16928875 PMC6674368

[B48] Rinberg D, Koulakov A, Gelperin A (2006) Speed-accuracy tradeoff in olfaction. Neuron 51:351–358. 10.1016/j.neuron.2006.07.013 16880129

[B49] Rolls ET, Kringelbach ML, de Araujo IET (2003) Different representations of pleasant and unpleasant odours in the human brain. Eur J Neurosci 18:695–703. 12911766 10.1046/j.1460-9568.2003.02779.x

[B50] Romo R, Hernández A, Zainos A, Lemus L, Brody CD (2002) Neuronal correlates of decision-making in secondary somatosensory cortex. Nat Neurosci 5:1217–1225. 10.1038/nn950 12368806

[B51] Schaefer AT, Margrie TW (2007) Spatiotemporal representations in the olfactory system. Trends Neurosci 30:92–100. 10.1016/j.tins.2007.01.001 17224191

[B52] Shusterman R, Smear MC, Koulakov AA, Rinberg D (2011) Precise olfactory responses tile the sniff cycle. Nat Neurosci 14:1039–1044. 10.1038/nn.2877 21765422 PMC13348895

[B53] Smear M, Shusterman R, O'Connor R, Bozza T, Rinberg D (2011) Perception of sniff phase in mouse olfaction. Nature 479:397–400. 10.1038/nature10521 21993623

[B54] Smear M, Resulaj A, Zhang J, Bozza T, Rinberg D (2013) Multiple perceptible signals from a single olfactory glomerulus. Nat Neurosci 16:1687–1691. 24056698 10.1038/nn.3519 PMC13445371

[B55] Smith DW, Gamble KR, Heil TA (2010) A novel psychophysical method for estimating the time course of olfactory rapid adaptation in humans. Chem Senses 35:717–725. 10.1093/chemse/bjq073 20696649

[B56] Steinmetz G, Pryor GT, Stone H (1970) Olfactory adaptation and recovery in man as measured by two psychophysical techniques. Percept Psychophys 8:327–330.

[B57] Stone H (1963) Determination of odor difference limens for three compounds. J Exp Psychol 66:466–473. 14082002 10.1037/h0045782

[B58] Stone H, Bosley JJ (1965) Olfactory discrimination and Weber's law. Percept Motor Skills 20:657–665. 10.2466/pms.1965.20.2.657 14279358

[B59] Stone H, Pryor GT, Steinmetz G (1972) A comparison of olfactory adaptation among seven odorants and their relationship with several physicochemical properties. Percept Psychophys 12:501–504. 10.3758/BF03210944

[B60] Stopfer M, Jayaraman V, Laurent G (2003) Intensity versus identity coding in an olfactory system. Neuron 39:991–1004. 12971898 10.1016/j.neuron.2003.08.011

[B61] Uchida N, Mainen ZF (2003) Speed and accuracy of olfactory discrimination in the rat. Nat Neurosci 6:1224–1229. 10.1038/nn1142 14566341

[B62] Wilson DA (1998) Habituation of odor responses in the rat anterior piriform cortex. J Neurophysiol 79:1425–1440. 9497422 10.1152/jn.1998.79.3.1425

[B63] Wilson DA, Stevenson RJ (2006) Learning to smell: olfactory perception from neurobiology to behavior. Baltimore: The John Hopkins UP.

[B64] Wilson RI, Mainen ZF (2006) Early events in olfactory processing. Annu Rev Neurosci 29:163–201. 10.1146/annurev.neuro.29.051605.112950 16776583

[B65] Wojcik PT, Sirotin YB (2014) Single scale for odor intensity in rat olfaction. Curr Biol 24:568–573. 24560575 10.1016/j.cub.2014.01.059

[B66] Yoshioka T, Gibb B, Dorsch AK, Hsiao SS, Johnson KO (2001) Neural coding mechanisms underlying perceived roughness of finely textured surfaces. J Neurosci 21:6905–6916. 11517278 10.1523/JNEUROSCI.21-17-06905.2001 PMC6763072

[B67] Zelano C, Sobel N (2005) Humans as an animal model for systems-level organization of olfaction. Neuron 48:431–454. 10.1016/j.neuron.2005.10.009 16269361

[B68] Zhou Z, Belluscio L (2012) Coding odorant concentration through activation timing between the medial and lateral olfactory bulb. Cell Rep 2:1143–1150. 10.1016/j.celrep.2012.09.035 23168258 PMC3513620

[B69] Zufall F, Leinders-Zufall T (2000) The cellular and molecular basis of odor adaptation. Chem Senses 25:473–481. 10944513 10.1093/chemse/25.4.473

